# Assessment of Ethanol-Induced Toxicity on iPSC-Derived Human Neurons Using a Novel High-Throughput Mitochondrial Neuronal Health (MNH) Assay

**DOI:** 10.3389/fcell.2020.590540

**Published:** 2020-11-05

**Authors:** Annika Zink, Josefin Conrad, Narasimha Swami Telugu, Sebastian Diecke, Andreas Heinz, Erich Wanker, Josef Priller, Alessandro Prigione

**Affiliations:** ^1^Department of Neuropsychiatry, Charité – Universitätsmedizin Berlin, Berlin, Germany; ^2^Max Delbrück Center for Molecular Medicine, Berlin, Germany; ^3^Department of General Pediatrics, Neonatology, and Pediatric Cardiology, Heinrich Heine University, Düsseldorf, Germany; ^4^University of Edinburgh and UK Dementia Research Institute, Edinburgh, United Kingdom

**Keywords:** iPSCs, neurons, mitochondria, ethanol, high-content screening, neuronal toxicity

## Abstract

Excessive ethanol exposure can cause mitochondrial and cellular toxicity. In order to discover potential counteracting interventions, it is essential to develop assays capable of capturing the consequences of ethanol exposure in human neurons, and particularly dopaminergic neurons that are crucial for the development of alcohol use disorders (AUD). Here, we developed a novel high-throughput (HT) assay to quantify mitochondrial and neuronal toxicity in human dopaminergic neuron-containing cultures (DNs) from induced pluripotent stem cells (iPSCs). The assay, dubbed mitochondrial neuronal health (MNH) assay, combines live-cell measurement of mitochondrial membrane potential (MMP) with quantification of neuronal branching complexity post-fixation. Using the MNH assay, we demonstrated that chronic ethanol exposure in human iPSC-derived DNs decreases MMP and neuronal outgrowth in a dose-dependent manner. The toxic effect of ethanol on DNs was already detectable after 1 h of exposure, and occurred similarly in DNs derived from healthy individuals and from patients with AUD. We next used the MNH assay to carry out a proof-of-concept compound screening using FDA-approved drugs. We identified potential candidate compounds modulating acute ethanol toxicity in human DNs. We found that disulfiram and baclofen, which are used for AUD treatment, and lithium caused neurotoxicity also in the absence of ethanol, while the spasmolytic drug flavoxate positively influenced MNH. Altogether, we developed an HT assay to probe human MNH and used it to assess ethanol neurotoxicity and to identify modulating agents. The MNH assay represents an effective new tool for discovering modulators of MNH and toxicity in live human neurons.

## Introduction

Ethanol is the most frequently abused drug worldwide and its excessive consumption is a leading risk factor for disability and death (Griswold et al., 2018). High ethanol consumption over time can lead to serious health and social problems, including the development of alcohol use disorder (AUD). AUD is among the most prevalent mental disorders in industrialized countries (Kessler et al., 2005). Once ingested, ethanol quickly distributes throughout the body and reaches the brain within minutes. Given this rapid and vast spread, ethanol can cause direct organ toxicity. Ethanol-induced neurotoxicity is particularly detrimental given that damaged neurons cannot be replaced. Within the central nervous system, ethanol exposure directly affects dopaminergic neurons, resulting in increased extracellular dopamine mainly in the striatum (Heinz et al., 2011), higher firing frequency and increased excitability (Trantham-Davidson and Chandler, 2015), as well as decreased dopamine synthesis and dopamine D2 receptor availability in AUD patients (Morikawa and Morrisett, 2010).

At the cellular level, an important role during ethanol intoxication may be played by mitochondria, crucial organelles that safeguard cellular homeostasis. Mitochondria provide energy in the form of ATP through oxidative phosphorylation (OXPHOS), which turns ADP into ATP by releasing the energy stored in the proton gradient also known as mitochondrial membrane potential (MMP) (Vafai and Mootha, 2012). Mitochondria are also crucial for cell death (Dyall et al., 2004). Selective MMP permeabilization activates caspase-driven apoptotic cell death through opening of the mitochondrial permeability transition pore (Tait and Green, 2013). MMP is thus an essential parameter for viable cells, since it is important for both ATP generation and initiation of apoptosis, and may serve as a marker of cell health (Chen, 1988). Various studies reported ethanol-induced toxicity in mitochondria located in the brain (Manzo-Avalos and Saavedra-Molina, 2010), including increased mitochondrial production of free radicals (Reddy et al., 2013), alterations in mitochondrial respiration and MMP (Haorah et al., 2013; Reddy et al., 2013), impairment of ATP production (Bustamante et al., 2012; Jung, 2015), and cell death induction (Lamarche et al., 2013; Jung, 2015).

The mechanisms underlying ethanol-induced toxicity in human brain cells remain largely unknown. Most investigations are based on animal models, which may not fully recapitulate the human disease, or on post-mortem tissues, which correlates more to an end-stage of AUD, or on brain imaging, which provides macroscopic data lacking information at the cellular and molecular level (Goldman, 2016). Given the lack of suitable human cell-based model systems for the assessment of neurotoxicity and drug discovery, our knowledge of compounds capable of counteracting ethanol toxicity in humans is limited.

Here, we used human induced pluripotent stem cells (iPSCs) to investigate the toxic consequences of ethanol in human neuronal cultures containing dopaminergic neurons. In order to assess human neurotoxicity in a quantitative and high-throughput (HT) manner, we devised a new assay that we named mitochondrial neuronal health (MNH) assay. The MNH assay is based on high-content imaging (HCI) and combines live-cell measurement of MMP with quantification of neuronal branching outgrowth. Using this assay, we demonstrated the acute and chronic effects of ethanol toxicity on MNH in human dopaminergic neuron-containing cultures (DNs), which we derived from iPSCs from healthy individuals and subjects with AUD. We also showed that the MNH assay can be used to perform compound screenings to identify drugs influencing MNH. Altogether, the newly developed MNH assay represents an effective HT tool for analyzing the cellular health of human iPSC-derived neurons and for discovering potential modulatory interventions.

## Results

### Development of the MNH Assay

**FIGURE 1:**
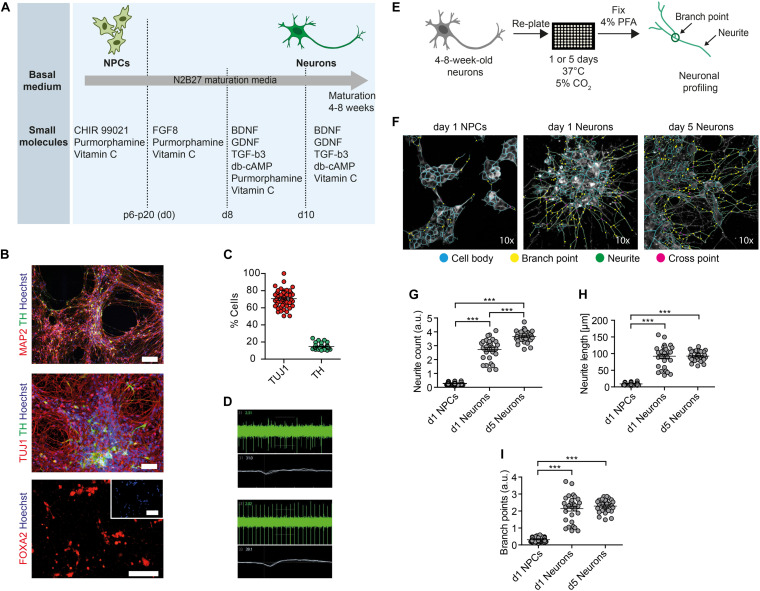
Generation of functional DNs and HCI-based neuronal profiling. **(A)** Schematics of the iPSC-based generation of NPCs and DNs, indicating the time-points of different media formulations. **(B)** 4- to 8-week-old DNs generated from control iPSCs (XM001 line) expressed the neuronal marker TUJ1 and MAP2, and the DA-specific markers FOXA2 and TH. Scale bar: 100 μm. **(C)** HCI-based quantification of control DNs (XM001) expressing TUJ1 and TH at 8 weeks of culture (mean ± SEM). Each dot represents mean values (% neuronal cells) from one well. **(D)** Representative recordings (spike plot and wave plot) of network-based electrophysiological properties of control DNs after 8 weeks in culture using MEA. **(E)** Schematic workflow of HCI-based neuronal profiling analysis. **(F)** Representative images of NPCs and DNs (at 4–8 weeks of differentiation) fixed after day 1, and of DNs fixed after day 5. Cell bodies depicted in blue, branch points in yellow, neurites in green, and cross points in magenta. All images were taken at 10× magnification. **(G–I)** Quantification of neurite count **(G)**, neurite length **(H)**, and branch points **(I)** in NPCs and 4- to 8-week-old DNs at day 1 and day 5 after re-plating (30 technical replicates for DNs and 60 technical replicates for NPCs; each dot represents the value obtained from one well; mean ± SEM; ***p < 0.001; one-way ANOVA followed by Dunnett’s multiple comparison test).

In order to assess the toxic effects of ethanol in human neurons, we first generated neural progenitor cells (NPCs) from healthy control-derived iPSCs (XM001 line) (Wang et al., 2018) and human embryonic stem cells (hESCs) (H1 line) (Thomson et al., 1998) using a small molecule-based protocol (Reinhardt et al., 2013). We then differentiated NPCs into post-mitotic neuronal cultures enriched for dopaminergic neurons, which we further refer to as dopaminergic neuron-containing cultures (DNs) (Figure 1A). The pluripotent stem cell-derived DNs expressed the neuron-specific marker MAP2 and the dopaminergic markers tyrosine hydroxylase (TH) and FOXA2 (Figure 1B). In the differentiated cultures, 75% of cells expressed the pan-neuronal marker beta tubulin-III (TUJ1) and 20% expressed TH (Figure 1C). These numbers are in accordance with previous protocols (Burbulla et al., 2017). We monitored neuronal network activity using micro-electrode array (MEA). We confirmed that the generated DNs were functional and exhibited multiple spontaneous spikes after 4–8 weeks in culture (Figure 1D).

We next established an HCI-based approach to assess mitochondrial function and neuronal growth capacity in DNs. We dubbed the method MNH assay. To assess neuronal branching capacity, we cultivated DNs for 4–8 weeks, re-plated them on the assay plate and kept them for 1 day or 5 days after plating. We then fixed the cultures and stained them with TUJ1 to visualize neuronal arborization (Figure 1E). Since TUJ1 staining does not allow to discriminate between axons and dendrites, we chose to refer to any projection from the cell body as neurite. In order to assess differences in neurite outgrowth, we compared DNs fixed 1 day or 5 days post-plating to NPCs fixed 1-day post-plating (Figure 1F). The MNH assay effectively captured and quantified differences in neuronal outgrowth. DNs at 5 days post-plating showed increased neurite count (Figure 1G), neurite length (Figure 1H), and branch points (Figure 1I) compared to neurons at day 1 post-plating or NPCs at day 1 post-plating. The cellular population also appeared more homogenous in day 5 DNs, as indicated by lower variability in the distribution of the replicates (Figures 1G–I).

**FIGURE 2:**
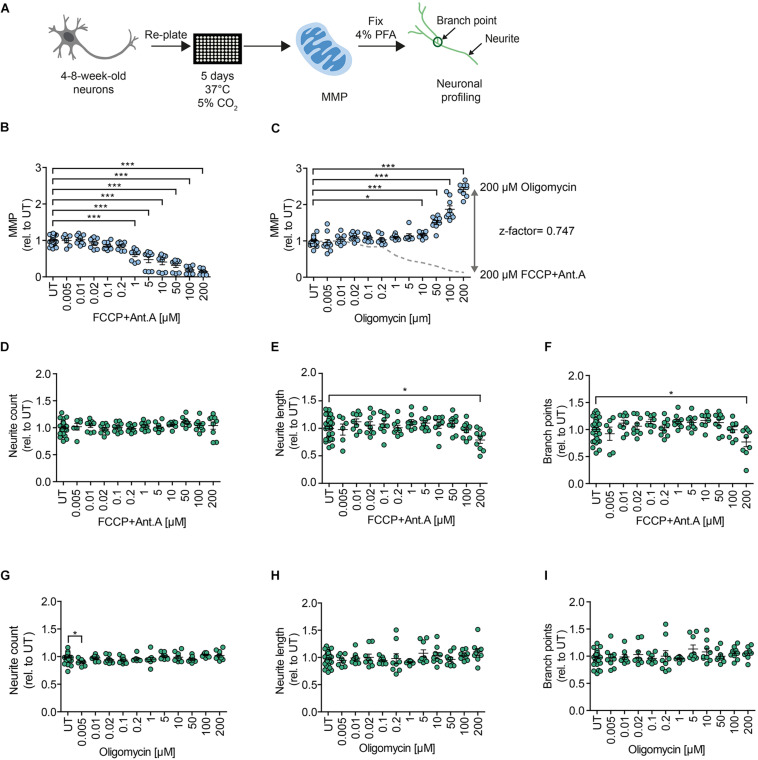
MNH assay development. **(A)** Schematics of the MNH assay. **(B)** Quantification of MMP in 4- to 8-week-old DNs derived from hESCs (H1 line) treated with increasing concentrations of FCCP and Ant.A (0.005–200 μM). Each dot represents the average of the values obtained from one well normalized to the corresponding untreated (UT) control exposed to the assay media only (*n* = 3 independent experiments; mean ± SEM; ***p < 0.001; one-way ANOVA followed by Dunnett’s multiple comparison test). **(C)** Quantification of MMP in 4- to 8-week-old DNs from control hESCs (H1 line) treated with increasing concentrations of oligomycin (0.005–200 μM; mean ± SEM; *p < 0.05, ***p < 0.001; one-way ANOVA followed by Dunnett’s multiple comparison test). Each dot represents the average of values obtained from one well normalized to the corresponding UT control exposed to the assay media only (*n* = 3 independent experiments). Assessment of the z-factor of 0.747 obtained at 200 μM FCCP and 200 μM Ant.A versus 200 μM Oligomycin suggesting an excellent assay. **(D–F)** Quantification of neurite count **(D)**, neurite length **(E)**, and branch points **(F)** in 4- to 8-week-old DNs from hESCs (H1 line) treated with increasing concentrations of FCCP and Ant.A (0.005–200 μM; mean ± SEM; *p < 0.05; one-way ANOVA followed by Dunnett’s multiple comparison test). Each dot represents values obtained from one well (*n* = 3 independent experiments), normalized to the corresponding UT controls exposed to the assay media only. **(G–I)** Quantification of neurite count **(G)**, neurite length **(H)**, and branch points **(I)** in 4- to 8-week-old DNs from hESCs (H1) treated with increasing concentrations of oligomycin (0.005–200 μM; mean ± SEM; *p < 0.05; one-way ANOVA followed by Dunnett’s multiple comparison test). Each dot represents values obtained from one well (*n* = 3 independent experiments), normalized to the corresponding UT controls exposed to the assay media only.

In order to assess MMP, we implemented an HCI assay that we previously established for iPSC-derived NPCs (Lorenz et al., 2017). We measured MMP using the potentiometric fluorescent dye tetramethylrhodamine methyl ester (TMRM), a lipophilic cation that accumulates in the mitochondrial matrix in proportion to the potential of the membrane. We re-plated 4- to 8-week-old DNs on the assay plate and kept them for 5 days before live-staining for MMP and subsequent neuronal branching quantification (Figure 2A). Stimulation with the OXPHOS uncoupler FCCP and the complex III inhibitor Antimycin A (Ant.A) provoked a dose-dependent decrease in the MMP (Figure 2B), whereas the ATPase inhibitor Oligomycin led to a dose-dependent increase in the MMP (Figure 2C). The MMP-modulating effects of the mitochondrial inhibitors are in line with previous works (Kalbácová et al., 2003; Li et al., 2014). The use of stimulating and inhibiting MMP modulators enabled us to determine the z-factor, a statistical indicator of HT bioassays that is considered excellent if 0.5 > *x* > 1 (Zhang et al., 1999). The MNH assay had a z-factor of 0.747, suggesting excellent features regarding reproducibility and robustness (Figure 2C). Branching complexity did not change upon exposure to FCCP and Ant.A at a concentration of up to 100 μM (Figures 2D–F), or to oligomycin at a concentration of up to 200 μM (Figures 2G–I). Taken together, the MNH assay was able to quantitatively identify changes in neuronal outgrowth capacity and in MMP-specific function.

### The MNH Assay Detects Chronic and Acute Ethanol-Induced Toxicity in Human Neurons

In alcohol-naïve individuals, blood alcohol concentrations (BACs) of 10–50 mM typically lead to sedation, motor incoordination, and cognitive impairment, and BACs of ≥100 mM cause strong sedation and can lead to coma or death (Adinoff et al., 1988). In contrast, BACs up to 300 mM have been reported in chronic alcohol consumers (Johnson et al., 1982), where 100–200 mM typically lead to sedation, anxiolysis, and hypnosis (Abrahao et al., 2017; Cui and Koob, 2017). For *in vitro* studies, the 10–100 mM ethanol range is therefore appropriate, and the addition of ethanol to cell cultures has been reported in the 1–500 mM range (Dolganiuc and Szabo, 2009). However, it is not clear at which concentrations ethanol can cause toxicity in human neuronal cultures.

**FIGURE 3:**
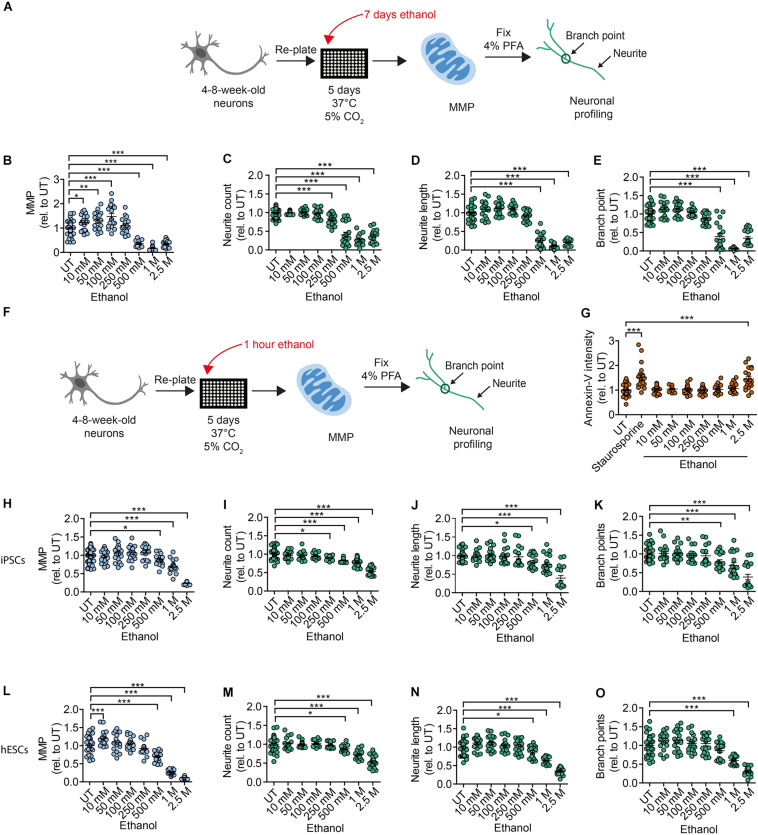
Chronic and acute ethanol exposure leads to decreased MMP and reduced branching complexity in iPSC-derived DNs. **(A)** Schematic of MNH assay workflow for chronic (7 days) ethanol exposure. **(B)** Quantification of the MMP in 4- to 8-week-old DNs from control iPSCs (XM001) exposed for 7 days (chronic) to increasing concentrations of ethanol (10 mM–2.5 M; mean ± SEM; *p < 0.05, **p < 0.01, ***p < 0.001; one-way ANOVA followed by Dunnett’s multiple comparison test). Each dot represents values obtained from one well (*n* = 3 independent experiments), normalized to the corresponding UT controls exposed to the assay media only. **(C–E)** Quantification of the neuronal branching complexity including neurite count **(C)**, neurite length **(D)**, and branch points **(E)** in 4- to 8-week-old DNs from control iPSCs (XM001) exposed to increasing concentrations of ethanol for 7 days (chronic treatment) (10 mM–2.5 M; mean ± SEM; ***p < 0.001; one-way ANOVA followed by Dunnett’s multiple comparison test). Each dot represents values obtained from one well (*n* = 3 independent experiments) normalized to the corresponding UT controls exposed to the assay media only. **(F)** Schematic of MNH assay workflow of ethanol exposure for 1 h (acute treatment). **(G)** Quantification of apoptosis via Annexin-V intensity on 4- to 8-week-old DNs from control iPSCs (XM001) exposed to ethanol for 1 h (mean ± SEM ***p < 0.005; one-way ANOVA followed by Dunnett’s multiple comparison test). Control wells included untreated DNs (UT) and DNs treated with 1 μM staurosporine for 1.5 h as a positive control for apoptosis. Each dot represents values obtained from one well (*n* = 4 independent experiments) normalized to UT controls exposed to the assay media only.**(H)** Quantification of MMP in 4- to 8-week-old DNs from control iPSCs (XM001) exposed to increasing concentrations of ethanol for 1 h (acute treatment; mean ± SEM; *p < 0.05, ***p < 0.001; one-way ANOVA followed by Dunnett’s multiple comparison test). Each dot represents values obtained from one well (*n* = 3 independent experiments) normalized to UT controls exposed to the assay media only. **(I–K)** Quantification of neuronal profiling including neurite count **(I)**, neurite length **(J)**, and branch points **(K)** in 4- to 8-week-old DNs from control iPSCs (XM001) exposed to increasing concentrations of ethanol for 1 h (acute exposure) (mean ± SEM; *p < 0.05, **p < 0.01, ***p < 0.001; one-way ANOVA followed by Dunnett’s multiple comparison test). Each dot represents values obtained from one well (*n* = 3 independent experiments) normalized to the corresponding UT controls exposed to assay media only. **(L)** Quantification of the MMP in 4- to 8-week-old DNs from hESCs (H1 line) exposed to increasing concentrations of ethanol for 1 h (acute exposure; mean ± SEM; ***p < 0.001; one-way ANOVA followed by Dunnett’s multiple comparison test). Each dot represents values obtained from one well (*n* = 3 independent experiments) normalized to the corresponding UT controls exposed to the assay media only. **(M–O)** Quantification of neuronal profiling including neurite count **(M)**, neurite length **(N)**, and branch points **(O)** in 4- to 8-week-old DNs from hESCs (H1 line) exposed to increasing concentrations of ethanol for 1 h (acute treatment; mean ± SEM; *p < 0.05, ***p < 0.001; one-way ANOVA followed by Dunnett’s multiple comparison test). Each dot represents values obtained from one well (*n* = 3 independent experiments) normalized to the corresponding UT controls exposed to the assay media only.

To address the toxic effects of ethanol on human DNs, we tested healthy DNs after 4–8 weeks of differentiation from iPSCs (XM001 line) and hESCs (H1 line). We exposed healthy DNs to different concentrations of ethanol in the media for 7 days (chronic exposure), with full media exchange every other day (Figure 3A). In our experimental setting, ethanol evaporation may occur inside the incubator during the exposure. This means, that the final ethanol concentration on the DNs may be lower than the one measured before the exposure. Chronic exposure to ethanol over 7 days increased MMP at ethanol concentrations of 10–100 mM and caused the MMP to collapse at ethanol concentrations ≥500 mM (Figure 3B). Ethanol concentrations higher than 250 mM also strongly impaired neurite outgrowth (Figures 3C–E).

We next investigated whether changes in MNH could also occur in DNs after short acute exposure to ethanol. We plated 4- to 8-week-old DNs generated from iPSCs (XM001) and hESCs (H1) on the assay plate, kept them for 5 days and then exposed them to ethanol for 1 h before the assay (Figure 3F). We observed the occurrence of apoptotic cell death (assessed by Annexin V staining) only in DNs exposed to 2.5 M ethanol concentration (Figure 3G). Hence, in our settings DNs retained viability even after exposure to relatively high concentrations of alcohol. Acute ethanol exposure led to a dose-dependent reduction of MMP starting at concentrations of 500 mM ethanol in DNs derived from iPSCs (Figure 3H) and hESC-derived DNs (Figure 3L). Acute ethanol exposure caused a dose-dependent reduction in neurite count starting at 250 mM ethanol in DNs derived from iPSCs (Figure 3I) and at 500 mM ethanol in DNs derived from hESCs (Figure 3M). Neurite length decreased starting from 500 mM ethanol in DNs derived from iPSCs (Figure 3J) and hESCs (Figure 3N). A dose-dependent decrease in branch points was observed at ≥500 mM ethanol in DNs derived from iPSCs (Figure 3K) and at ≥1 M ethanol in DNs derived from hESCs (Figure 3O).

Altogether, using the MNH assay to quantify ethanol-induced neurotoxicity, we determined that both acute and chronic exposure to ethanol negatively affected human MNH. MMP and branching defects occurred for ethanol concentrations lower than those needed to elicit apoptotic cell death. The findings suggest that ethanol-induced toxicity in human neurons happens very rapidly, and that the MNH assay is able to capture early toxic states that are not yet causing widespread cell death.

### Neurons From AUD Patients Recapitulate Acute Ethanol-Induced Neurotoxicity

**FIGURE 4:**
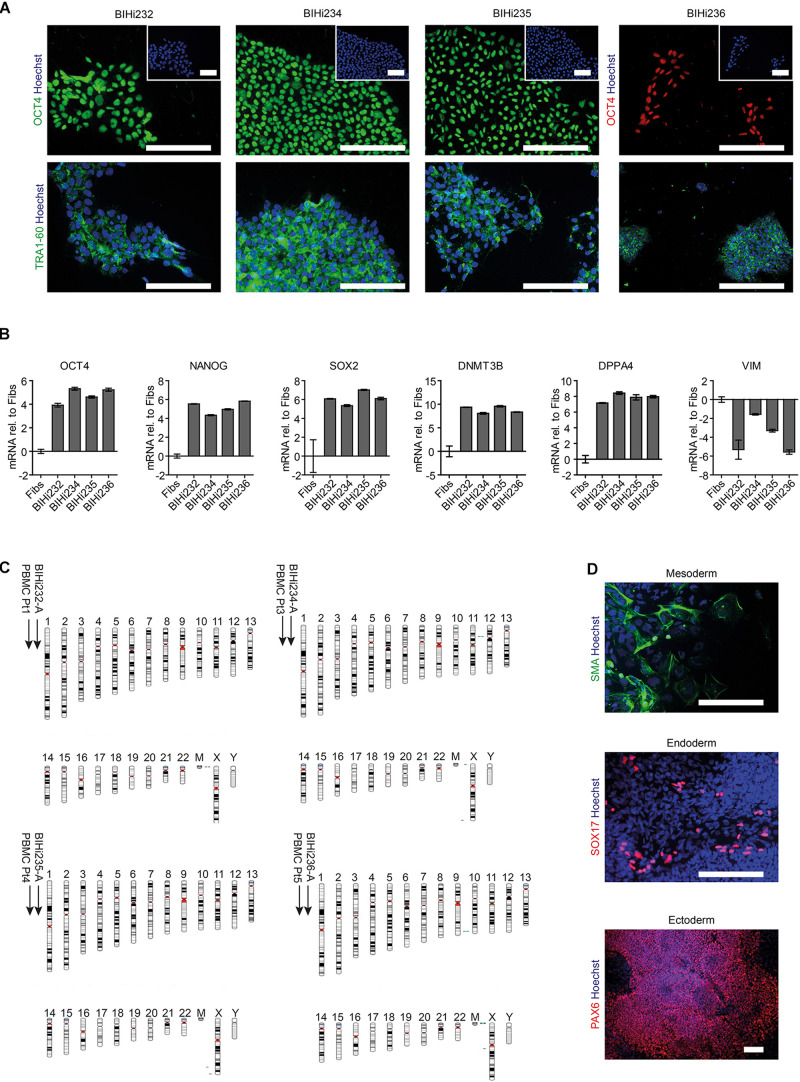
Generation of patient-specific AUD-iPSCs. **(A)** Representative immunostaining images of pluripotency-associated markers OCT4 and TRA1-60 in AUD-patient derived iPSCs (BIHi232, BIHi234, BIHi235, and BIHi236). We counterstained the cells using Hoechst. Scale bar: 100 μm. **(B)** Quantitative real-time RT-PCR analysis of pluripotency-associated markers in AUD-iPSCs (BIHi232, BIHi234, BIHi235, and BIHi236) relative to fibroblasts (Fibs; CON2). Relative transcript levels of each gene were calculated based on the 2^–ΔΔCT^ method. Data were normalized to the housekeeping gene *GAPDH* and are presented as mean LOG2 ratios in relation to fibroblasts (mean ± SD). **(C)** Single nucleotide polymorphism (SNP)-based virtual karyotype of AUD-iPSCs. We compared the karyotype of the iPSCs to the corresponding parental cells (peripheral blood mononuclear cells, PBMCs). We did not see any larger areas of insertions or deletions. Green: area with genomic gain; red: with genomic loss; gray: area with loss of heterozygosity. Pt, patient. **(D)** Representative immunostaining images of AUD-iPSCs differentiated into cells belonging to the three germ layers: mesoderm (smooth muscle actin, SMA), endoderm (SOX17), and ectoderm (PAX6). Scale bar: 100 μm.

**FIGURE 5:**
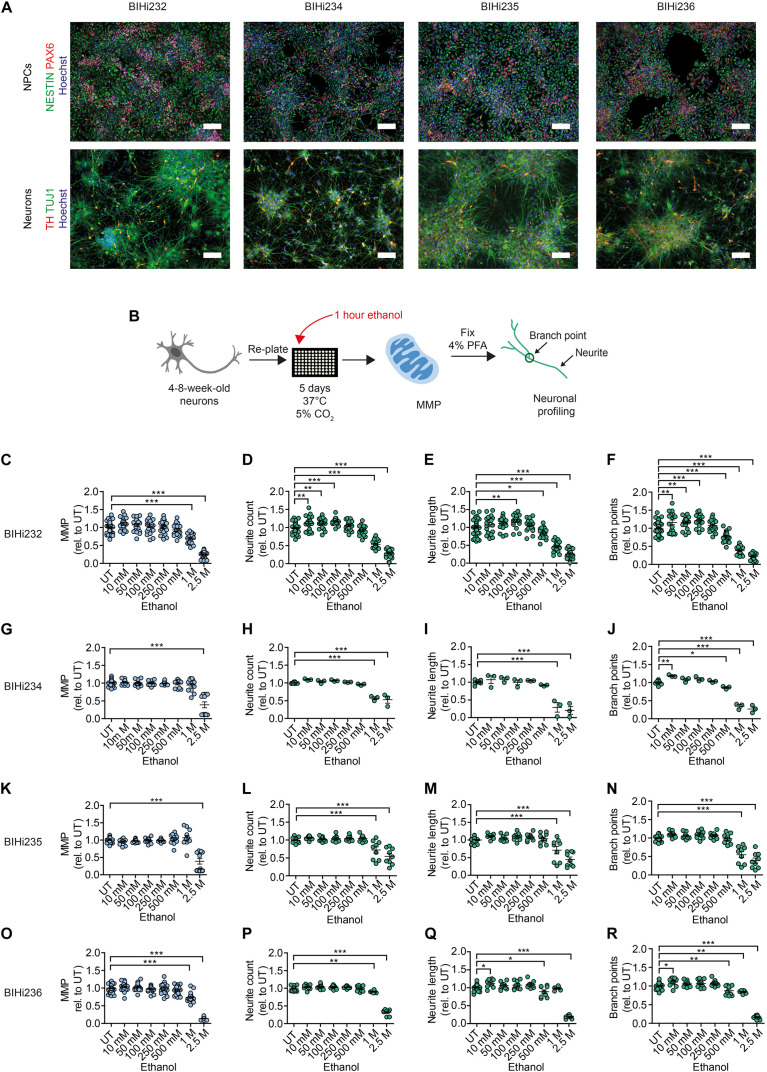
Acute ethanol exposure reduces MMP and neuronal outgrowth in AUD-DNs. **(A)** Above: representative immunostaining images of NPC-associated markers NESTIN and PAX6 counterstained with Hoechst in NPCs from AUD-iPSCs (BIHi232, BIHi234, BIHi235, and BIHi236). Scale bar: 100 μm. Below: representative immunostaining images of neuron-associated marker TUJ1 and dopaminergic neuron-associated marker TH counterstained with Hoechst in AUD-DNs (BIHi232, BIHi234, BIHi235, and BIHi236). Scale bar: 100 μm. **(B)** Schematic of MNH assay workflow of ethanol exposure for 1 h (acute exposure) on AUD-DNs. **(C–R)** MNH assay-based quantification of MMP **(C,G,K,O)** and branching complexity **(D–F,H–J,L–N,P–R)** in 4- to 8-week-old AUD-DNs (**C–F** BIHi232; **G–J** BIHi234; **K–N** BIHi235; **O–R** BIHi236) exposed to increasing concentrations of ethanol for 1 h (mean ± SEM; *p < 0.05, **p < 0.01, ***p < 0.001; one-way ANOVA followed by Dunnett’s multiple comparison test). Each dot represents values obtained from one well (*n* = 3 independent experiments) normalized to the corresponding UT controls.

Next, we aimed to address ethanol-induced neurotoxicity in DNs derived from individuals diagnosed with AUD according to DSM-5 and ICD-10 (alcohol dependence). Using Sendai virus-mediated reprogramming, we generated iPSCs from four AUD patients: BIH232, BIH234, BIH235, and BIH236. All AUD-iPSCs expressed the pluripotency-associated protein markers OCT4 and TRA1-60 (Figure 4A). Compared to somatic fibroblasts, AUD-iPSCs exhibited higher mRNA expression of the pluripotency-associated gene markers *OCT4, NANOG, SOX2, DNMT3B*, and *DPPA4*, and lower expression of the fibroblast-associated gene marker *VIM* (Figure 4B). The AUD-iPSCs had normal karyotypes (Figure 4C) and were pluripotent as they were capable of generating cells belonging to the three germ layers: endoderm, mesoderm, and ectoderm (Figure 4D). We then differentiated AUD-iPSCs into NPCs and DNs. AUD-NPCs expressed the NPC-associated markers NESTIN and PAX6 (Figure 5A). AUD-DNs expressed the pan-neuronal protein marker TUJ1 and the dopaminergic neuron marker TH (Figure 5A).

We used the MNH assay to quantify changes in MMP and neuronal arborization in AUD-DNs following 1 h of acute ethanol exposure (Figure 5B). Acute ethanol exposure decreased MMP in AUD-DNs as observed in healthy DNs, but only at high concentrations of 1 M and 2.5 M ethanol for lines BIH232 (Figure 5C) and BIH236 (Figure 5O), and 2.5 M ethanol for lines BIH234 (Figure 5G) and BIH235 (Figure 5K). Acute ethanol exposure also caused a dose-dependent reduction of neurite outgrowth requiring higher ethanol concentrations in AUD-DNs than in healthy DNs, starting at 1 M ethanol for neurite count (Figures 5D,H,L,P), 500 mM–1 M ethanol for neurite length (Figures 5E,I,M,Q), and 500 mM–1 M ethanol for branch points (Figures 5F,J,N,R).

Taken together, acute ethanol exposure negatively affected MNH in AUD-derived DNs. The individual AUD lines showed some level of heterogeneity, which may reflect different susceptibility to alcohol toxicity in different subjects.

### MNH-Based Proof-of-Concept Compound Screening Identified Modulators of Ethanol Neurotoxicity

**FIGURE 6:**
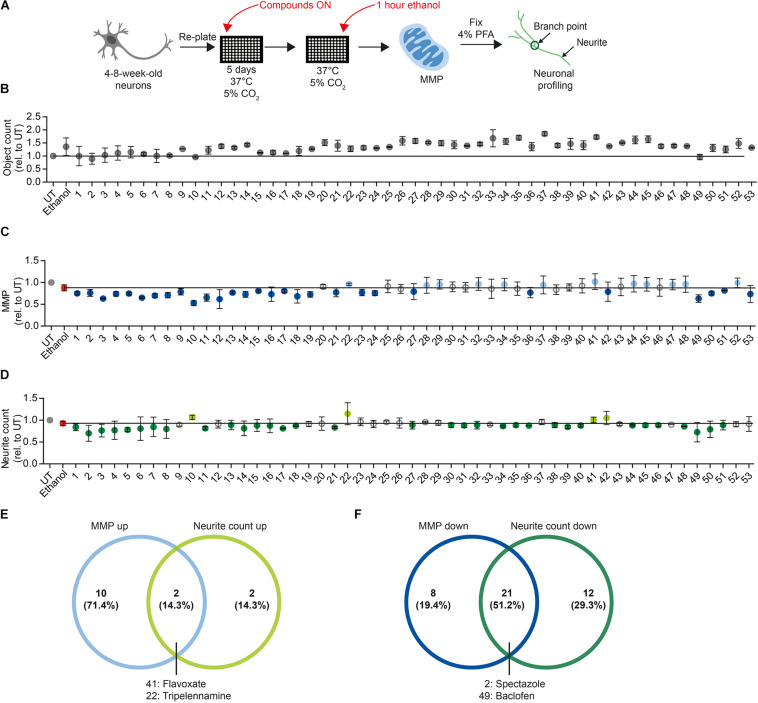
Proof-of-concept compound screening in human DNs exposed to ethanol. **(A)** Schematic MNH assay workflow for compound screening. **(B)** MNH assay-based quantification of object count of DNs from control iPSCs (XM001 line) after ON treatment with 48 FDA-approved drugs (Selleckchem, z65122) and 5 AUD drugs with subsequent exposure to 1 M ethanol for 1 h (acute exposure; mean ± SD; *n* = 2 independent experiments). Values from each experiment were normalized to the corresponding UT controls. The black line marks the mean of UT control. **(C,D)** MNH assay-based quantification of MMP **(C)** and neuronal profiling (**D**; neurite count) after ON treatment with 48 FDA-approved drugs out of the Selleckchem library (z65122) and 5 AUD drugs with subsequent exposure to 1 M ethanol for 1 h (acute exposure). Values from each experiment were normalized to the corresponding UT controls (mean ± SD; *n* = 2 independent experiments). The black line marks the mean value of ethanol exposure (1 M, red dot). Gray circles mark the compounds that displayed no change in MMP or neurite counts. Light blue dots in panel **(C)** mark positive hit compounds that increased MMP of more than 0.5 times the SD of neurons only exposed to ethanol. Dark blue dots in panel **(C)** mark negative hit compounds that decreased the MMP more than 0.5 times the SD of neurons only exposed to ethanol. Light green dots in panel **(D)** mark positive hit compounds that increased neurite count of more than 0.5 times the SD of neurons only exposed to ethanol. Dark green dots in panel **(D)** mark negative hit compounds that decreased neurite count of more than 0.5 times the SD of neurons only exposed to ethanol. **(E)** Venn diagram depicting the compounds that increased MMP (“MMP up”) and/or neuronal outgrowth (“neurite count up”) in ethanol-exposed DNs. **(F)** Venn diagram depicting the compounds that decreased the MMP (“MMP down”) and/or the neuronal outgrowth (“neurite count down”) in ethanol-exposed DNs.

We next sought to determine whether the MNH assay could be used to carry out compound screenings to identify modulators of neurotoxicity. For screening, we used DNs derived from control iPSCs (XM001 line), which are more sensitive to ethanol than AUD-DNs. We focused our attention on acute ethanol exposure, which may still be potentially reversible. Using a library containing 700 FDA-approved drugs (Selleckchem # z65122), we selected 48 compounds that are approved for clinical use in the context of brain diseases (compound numbers 1–48; Figures 6B–D and Appendix Table 1). Additionally, we included five drugs that are used for the treatment of AUD patients (compound numbers 49–53; Figures 6B–D and Appendix Table 1).

We plated 4- to 8-week-old DNs and cultured them for 5 days. We treated DNs overnight (ON) with 0.5 μM of each of the 53 compounds in a proof-of-concept experiment. We then exposed DNs to 1 M ethanol for 1 h and performed the MNH assay (Figure 6A). Total object count, obtained by quantifying Hoechst-stained nuclei, showed no significant changes in cell number following the compound treatments, suggesting that we could confidently compare treated samples to untreated samples (Figure 6B). As baseline for comparisons, we used DNs treated for 1 h with 1 M ethanol that were not pre-exposed to any compound (Figures 6C,D, red dot and black horizontal line). We defined a compound as a “positive hit” for MMP or neurite count when the compound led to values that were at least 0.5 times higher than the standard deviation (SD) of neurons only exposed to ethanol (Figures 6C,D, light blue dots and light green dots). We defined a compound as a “negative hit” for MMP or neurite count when the compound led to values that were at least 0.5 times lower than the SD of neurons only exposed to ethanol (Figures 6C,D, dark blue dots and dark green dots).

Twelve positive hit compounds increased the MMP in the presence of ethanol (Figure 6C, light blue dots), and four positive hit compounds increased the number of neurites in the presence of ethanol (Figure 6D, light green dots). Two positive hit compounds ameliorated both the MMP and neurite count, namely flavoxate and tripelennamine (Figure 6E).

Twenty-nine negative hit compounds decreased the MMP further than ethanol alone (Figure 6C, dark blue dots), and 33 negative hit compounds decreased the neurite count further (Figure 6D, dark green dots). Twenty-one negative hit compounds decreased both the MMP and neurite count, suggesting enhanced neurotoxicity in addition to ethanol toxicity (Figure 6F). Among these 21 negative hit compounds, we identified spectazole and baclofen (Figure 6F).

**FIGURE 7:**
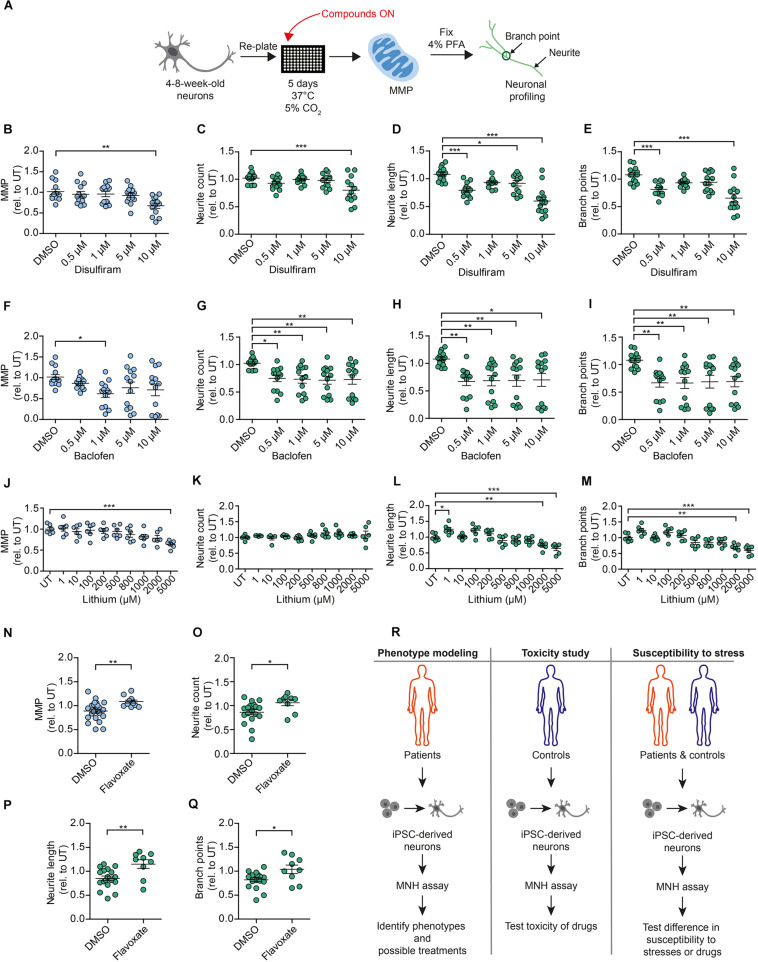
Compounds modulating neuronal toxicity in human DNs. **(A)** Schematic MNH assay workflow for neuronal toxicity. **(B–E)** MNH assay-based quantification of MMP **(B)** and neuronal profiling **(C–E)** including neurite count **(C)**, neurite length **(D)**, and branch points **(E)** after ON treatment of 4- to 8-week-old DNs from control iPSCs (XM001) with increasing concentrations of disulfiram (*n* = 3 independent experiments; mean ± SEM; *p < 0.05, **p < 0.01; ***p < 0.001; one-way ANOVA followed by Dunnett’s multiple comparison test). **(F–I)** MNH assay-based quantification of MMP **(F)** and neuronal profiling **(G–I)** including neurite count **(G)**, neurite length **(H)**, and branch points **(I)** after ON treatment of 4- to 8-week-old DNs from control iPSCs (XM001) with increasing concentrations of baclofen (*n* = 3 independent experiments; mean ± SEM; *p < 0.05, **p < 0.01; one-way ANOVA followed by Dunnett’s multiple comparison test). **(J–M)** MNH assay-based quantification of MMP **(J)** and neuronal profiling **(K–M)** including neurite count **(K)**, neurite length **(L)**, and branch points **(M)** after ON treatment of 4- to 8-week-old DNs from control iPSCs (XM001) with increasing concentrations of lithium (*n* = 3 independent experiments; mean ± SEM; *p < 0.05, **p < 0.01; ***p < 0.001; one-way ANOVA followed by Dunnett’s multiple comparison test). **(N–Q)** MNH assay-based quantification of MMP **(N)** and neuronal profiling **(O–Q)** including neurite count **(O)**, neurite length **(P)**, and branch points **(Q)** after ON treatment of 4- to 8-week-old DNs from control iPSCs (XM001) with 1 μM flavoxate (*n* = 3 independent experiments; mean ± SEM; *p < 0.05, **p < 0.01; one-way ANOVA followed by Dunnett’s multiple comparison test). **(R)** Schematics of potential applications of the MNH assay.

We then aimed to assess whether the modulators of MNH identified in the screening could exert a modulatory effect on DNs independent of ethanol. We used 4- to 8-week-old DNs from control iPSCs (XM001 line), re-plated them, and kept them for 5 days before ON treatment with the compounds (Figure 7A). As negative hits from the screening, we focused on drugs that are commonly employed in the context of AUD, such as disulfiram and baclofen. In the screening, disulfiram decreased the MMP and baclofen decreased both the MMP and neurite count in ethanol-exposed neurons (Figures 6C,D, baclofen = compound #49, disulfiram = compound #53). Using different biological replicates and several drug doses, we found that 0.5–10 μM disulfiram negatively affected the MMP (Figure 7B) and neuronal outgrowth (Figures 7C–E) in the absence of ethanol. Baclofen also negatively affected the MMP and neuronal outgrowth of DNs at concentrations between 0.5 and 10 μM in the absence of ethanol (Figures 7F–I). We then assessed the effect of lithium carbonate (lithium), which was not included in the original screen, on the MNH of DNs. Lithium is used for bipolar disorders (Young, 2009), and it has been considered for the treatment of AUD (Fawcett et al., 1987; Clark and Fawcett, 1989). We found that lithium also negatively affected the MMP of human DNs at concentrations between 2 and 5 mM in the absence of ethanol (Figure 7J). Although neurite count was unaffected (Figure 7K), lithium treatment significantly decreased neurite length (Figure 7L) and branch points (Figure 7M) of human DNs at concentrations between 2 and 5 mM in the absence of ethanol.

As a positive hit compound from the screening, we selected flavoxate. DNs treated with 1 μM flavoxate showed increases of the MMP (Figure 7N) and neurite arborization (Figures 7O–Q) compared to DNs only treated with DMSO. Taken together, these experiments confirmed some of the negative and positive hits of the screening and suggested that by identifying compounds counteracting neurotoxicity, it may be possible to discover general modulators of MNH in humans.

## Discussion

In this study, we developed a novel method called the MNH assay to assess the cellular health of human neurons derived from human pluripotent stem cells. We employed the MNH assay to dissect the neurotoxicity induced by ethanol on neuronal cultures containing dopaminergic neurons, which have been implicated in the development of AUD. Previous iPSC-based studies assessed alcohol-mediated transcriptional changes in NMDA receptor expression (Lieberman et al., 2012), in GABA receptor expression (Lieberman et al., 2018), and in the expression of genes involved in cholesterol homeostasis, notch signaling, and cell cycle (Jensen et al., 2019). Ethanol exposure for 1 day or 7 days was found to negatively influence the generation of mature neurons from NPCs (De Filippis et al., 2016). However, the functional effects of ethanol on mitochondrial neuronal function and neurite outgrowth have not yet been investigated. Using the MNH assay, we show that acute exposure to ethanol is sufficient to cause loss of MNH, similar to what happens after 7 days of chronic ethanol exposure. Importantly, acute ethanol-induced mitochondrial and neuronal toxicity occurred in both DNs derived from healthy individuals and from subjects diagnosed with AUD. Interestingly, the latter were less sensitive to ethanol toxicity. In contrast to previous studies, which did not focus on functional readouts, the defects in human neurons developed at relatively high concentrations of ethanol. Nonetheless, these concentrations did not induce widespread cell death, and the MNH assay was able to capture an early state of cellular toxicity that may still be reversible. Hence, the MNH assay may serve as a platform for identifying early modulators of neurotoxicity in humans.

Using the MNH assay, we identified potential modulators of ethanol-induced neurotoxicity. In a proof-of-concept screening of the 53 FDA-approved drugs that are in clinical use for brain diseases, flavoxate had positive effects on mitochondrial health and neurite outgrowth capacity. These positive effects also occurred in the absence of ethanol exposure. Conversely, 21 compounds showed a potential worsening effect in the presence of ethanol. Further studies should be carried out to confirm the exploratory results and determine the pharmacokinetic and pharmacodynamic characteristics of the hit candidates. Our findings suggest that the MNH assay could be used for various applications, including disease-specific studies, neurotoxicity studies, and studies aimed to assess the individual neuronal susceptibility to different stimuli (Figure 7R).

Most drugs of abuse, including alcohol, have significant neurotoxic effects (Soleimani et al., 2016). Understanding the level of neurotoxicity caused by ethanol may enable the development of protective drugs to reduce the risk of developing neurodegenerative effects as consequence of excessive alcohol consumption. Interestingly, two drugs commonly employed in subjects with AUD, disulfiram and baclofen, negatively modulated MNH. Their negative effects also occurred in the absence of ethanol. Treatment with lithium, another drug that can be employed in the context of AUD, also worsened MNH in the absence of ethanol. These latter data are in agreement with previous studies reporting toxic consequences of lithium treatment (Lang and Davis, 2002; Mignarri et al., 2013; Shah et al., 2015). Altogether, our findings on AUD drugs warrant further exploration and raise concerns with respect to the potentially enhanced toxicity of drugs when used in concert with alcohol intake, which can increase neurotoxicity. In-depth analyses are warranted to confirm the functional consequences of various AUD treatments on human neuronal health.

The assessment of possible toxic effects of compounds is of high importance in the drug development process. Neurotoxicity testing commonly relies on *in vivo* animal studies that are expensive and may not fully recapitulate the toxicity profile of humans (Hartung, 2008; Schwartz et al., 2015). Recent studies used MEA to investigate the effect of compounds on spontaneous neuronal activity and network activity (Zwartsen et al., 2018). Various HT screening methods have also been established (Saunders et al., 2014; Iannetti et al., 2015; Gelles and Chipuk, 2016; Daniele et al., 2017; Kepiro et al., 2018; Arias-Fuenzalida et al., 2019; Varkuti et al., 2020). Nonetheless, there is a need for additional *in vitro* systems centered on human iPSC derivatives in order to capture different aspects of human neurotoxicity (Schmidt et al., 2017). We suggest the MNH assay as a novel HT assay centered on human pluripotent stem cell-derived neurons that can be further multiplexed with additional imaging-based techniques, including for example the addition of an apoptotic dye to quantify cell death (Saunders et al., 2014; Gelles and Chipuk, 2016). iPSC-derived 3D neural cultures may also be employed to assess the neurotoxic potential of drugs and environmental toxicants (Sirenko et al., 2019). Therefore, future implementations should aim to adapt the MNH assay to 3D culture systems, such as iPSC-derived brain organoids (Menacho and Prigione, 2020).

Taken together, we developed a novel assay that enabled us to capture the ethanol-induced toxicity in human DNs and to identify potential small molecule modulators. We propose the MNH assay as a tool for the evaluation of human neuronal health and for conducting HT drug discovery and drug toxicity studies.

## Materials and Methods

### Generation of iPSCs From PBMCs

This study was approved by the ethics committee of the Charité – Universitätsmedizin Berlin (EA1/206/15). Patient peripheral blood mononuclear cells (PBMCs) were obtained after informed consent. In brief, blood collections were performed using standard, 8 ml Vacutainer Cell Processing Tubes (both sodium citrate and sodium heparin-based tubes are acceptable; BD Biosciences; Franklin Lakes, NJ, United States). Samples were processed within 24 h after blood collection by collecting the PBMC-containing upper phase and washed with ice-cold PBS. Cells were either frozen down or used directly for enrichment of the erythroblast population. In brief, cell pellet of 1 × 10^6^–2 × 10^6^ PBMCs was resuspended in 2 ml erythroblast cell expansion media containing basal blood media Stempo34 SFM (Thermo Fisher) supplemented with 1x Stempo34 nutrient supplement (Thermo Fisher), 1x glutamine (Thermo Fisher) and cytokines such as 20 ng/ml IL3 (PeproTech), 20 ng/ml IL6 (PeproTech), 100 ng/ml Flt3 (PeproTech), 100 ng/ml SCF (PeproTech), 2 U/ml erythropoietin (EPO, Millipore) and plated in a standard 12-well plate (2 mL/well). The media was changed every other day and after 4 to 6 days a significant enrichment of highly proliferating erythroblast population was observed. This highly proliferative cell population was used for the reprogramming. Erythroblast cells from PBMCs were reprogrammed using the Cytotune^®^-iPS 2.0 Sendai Reprogramming Kit according to the manufacturer’s instructions. In brief, cells were transduced with CytoTune 2.0 Sendai virus coding for *OCT3/4*, *KLF4*, *SOX*2, and *MYC* genes with recommended MOI in 1 ml of erythroblast cell expansion media containing 10 μg/ml polybrene (Sigma). After 24 h of transduction the virus was removed, and cells were cultured for further 2 days in erythroblast cell expansion media. From day 1 to 3 the cells were cultured in repro media 1 consisting of erythroblast cell expansion media, 200 μM/L sodium butyrate (NaB, Stem Cell Technologies) and 50 μg/ml ascorbic acid (Sigma). On day 3 after transduction 1 × 10^5^ cells were plated onto mouse embryonic fibroblast (MEFs) layer or vitronectin coated plate in repro media 1. From day 4 until day 7, the media was changed with repro media 2 containing basal blood media, NaB and ascorbic acid without cytokines. On day 7 the media was switched to repro media 2 and E7 reprogramming media (1:1) containing E6 basal media (Thermo Fisher) with NaB, ascorbic acid and 100 ng/ml FGF2 (PeproTech) and the same was used every day until day 13. From day 13 onward the cells were cultured in E8 life (Thermo Fisher) until day 20. Emerging colonies were picked 3 weeks after the transduction and tested for remaining viral RNA expression using RT-PCR by checking the expression of Sendai viral reprogramming particles using primers specific to Sendai virus.

### iPSC Culture

Control iPSCs were previously generated using episomal plasmids and described as XM001 (Wang et al., 2018). hESC line H1 was purchased from WiCell and was used in accordance with the German license of AP issued by the Robert Koch Institute (AZ: 3.04.02/0077-E01). All PSCs were cultivated on Matrigel (BD Bioscience)-coated plates using: StemMACS iPS-Brew XF medium (Miltenyi Biotec GmbH, #130-104-368), supplemented with 0.1 mg/ml Pen/Strep (Thermo Fisher Scientific) and MycoZap [1x] (Lonza). We routinely monitored against mycoplasma contamination using PCR. 10 μM ROCK inhibitor (Enzo, ALX-270-333-M005) was added after splitting to promote survival. PSC cultures were kept in a humidified atmosphere of 5% CO_2_ at 37°C and 5% oxygen. All other cultures were kept under atmospheric oxygen condition. Pluripotency of the generated lines was confirmed following previously published procedures (Prigione et al., 2010) using *in vitro* embryoid bodies (EB)-based differentiation. We reprogrammed patient PBMCs using Sendai viruses.

### Karyotype Analysis

Briefly, genomic DNA was isolated using the DNeasy blood and tissue kit (Qiagen, Valencia, CA, United States) and samples were analyzed using the human Illumina OMNI-EXPRESS-8v1.6 BeadChip. First, the genotyping was analyzed using GenomeStudio 1 genotyping module (Illumina). Thereafter, KaryoStudio 1.3 (Illumina) was used to perform automatic normalization and to identify genomic aberrations utilizing settings to generate B-allele frequency and smoothened Log R ratio plots for detected regions. The stringency parameters used to detect copy number variations (CNVs) were set to 75 kb (loss), 100 kb (gain) and CN-LOH (loss of heterozygosity) regions larger than 3 MB.

### Differentiation of NPCs and DNs

We obtained NPCs and DNs using a previously published protocol (Reinhardt et al., 2013). Briefly, PSCs were detached from Matrigel-coated plates using StemPro^®^ Accutase^®^ Cell Dissociation Reagent (accutase; Thermo Fisher) and the collected sedimented cells were transferred into low-attachment petri dishes and kept for 2 days in ES-based medium containing: KO-DMEM [1x] (Gibco), KO-SR [1x] (Gibco), NEAA [1x] (Gibco), 2 mM L-glutamine (Gibco), 0.1 mg/ml Pen/Strep (Gibco), 1 mM pyruvate (Gibco) and MycoZap [1x] (Lonza), supplemented with 0.5 μM purmorphamine (PMA, Millipore), 3 μM CHIR 99021 (CHIR, Caymen Chemical), 10 μM SB-431542 (MACS Miltenyi) and 1 μm dorsomorphin (Sigma). From day 2 to day 4, the media was switched to N2B27 medium containing: Neurobasal:DMEM/F12 [1:1], N2 [1:200], B27 without vitamin A [1:100], 2 mM L-glutamine, 0.1 mg/ml Pen/Strep, 1 mM Pyruvate and MycoZap [1x] supplemented with 0.5 μM PMA, 3 μM CHIR 99021, 10 μM SB-431542 and 1 μM dorsomorphin. On day 4 the media was switched to the final maturation media consisting of N2B27 medium supplemented with 3 μM CHIR 99021, 0.5 μM PMA and 150 μM vitamin C (Sigma Aldrich). On day 6, the suspended cells were transferred onto Matrigel-coated well plates and kept in the maturation media with media exchange every 2 days. NPCs were maintained on this media without ROCK inhibitor and used for experiments between passage 7 and 20. For neuronal differentiation, we used NPCs between passage 7 and 13. To initiate the differentiation, the media was changed to N2B27 (B27 with vitamin A) medium with addition of 200 μM vitamin C, 100 ng/mL FGF8 (R&D Systems) and 1 μM PMA. After 8 days, the media condition was replaced with N2B27 (B27 with vitamin A) media supplemented with 200 μM vitamin C, 0.5 μM PMA, 500 μM cAMP (StemCells), 10 ng/mL BDNF (MACS Miltenyi), 10 ng/mL GDNF (MACS Miltenyi) and 1 ng/mL TGFbeta3 (MACS Miltenyi). On day 9, cells were split with accutase and seeded on Matrigel-coated plates in N2B27 (B27 with vitamin A) medium supplemented with 200 μM vitamin C, 500 μM cAMP, 10 ng/mL BDNF, 10 ng/mL GDNF, and 1 ng/mL TGFbeta3. The media was changed every 3–4 days and the differentiated cells were kept in culture for 4 weeks up to 8 weeks. 10 μM ROCK inhibitor (Enzo, ALX-270-333-M005) was always added after splitting to promote survival.

### PCR Analyses

Gene expression analysis was performed by quantitative real-time RT-PCR (qRT-PCR) using SYBR Green PCR Master Mix and the ViiA^TM^ 7 Real-Time PCR System (Applied Biosystems). For each target gene, cDNA samples and negative controls were measured in triplicates using 384-Well Optical Reaction Plates (Applied Biosystems). Relative transcript levels of each gene were calculated based on the 2^–ΔΔCT^ method. Data were normalized to the housekeeping gene *GAPDH* and are presented as mean LOG2 ratios in relation to control cell lines. Primers were for OCT4 F: GTGGAGGAAGCTGACAACAA and R: ATTCTCCAGGTTGCCTCTCA, for NANOG F: CC TGTGATTTGTGGGCCTG and R: GACAGTCTCCGTGTG AGGCAT, for SOX2 F: GTATCAGGAGTTGTCAAGGCAGAG and R: TCCTAGTCTTAAAGAGGCAGCAAAC, for DNM T3B F: GCTCACAGGGCCCGATACTT and R: GCAGTCCTG CAGCTCGAGTTTA, for DPP4 F: TGGTGTCAGGTGGTGTG TGG and R: CCAGGCTTGACCAGCATGAA, for VIM F: GGAGCTGCAGGAGCTGAATG and R: GACTTGCCTTGGC CCTTGAG, for GAPDH F: CTGGTAAAGTGGATATTGT TGCCAT and R: TGGAATCATATTGGAACATGTAAACC.

### Immunostaining

Cells grown on Matrigel-coated coverslips were fixed with 4% paraformaldehyde (PFA, Science Services) for 15 min at room temperature (RT) and washed two times with PBS. For permeabilization, cells were incubated with blocking solution containing 10% normal donkey serum (DNS) and 1% Triton X-100 (Sigma-Aldrich) in PBS with 0.05% Tween 20 (Sigma-Aldrich) (PBS-T) for 1 h at RT. Primary antibodies were diluted in blocking solution and incubated overnight at 4°C on a shaker. Primary antibodies were used as follows: PAX6 (Covance, 1:200), SOX2 (Santa Cruz, 1:100), TUJ1 (Sigma-Aldrich, 1:3000), OCT4 (Santa Cruz, 1:300), TRA-1-60 (Millipore, 1:200), MAP2 (Synaptic System, 1:100), NANOG (R&D Systems, 1:200), SMA (DakoCytomation, 1:200), SOX17 (R&D Systems, 1:50), TH (Millipore, 1:300), FOXA2 (Sevenhills, 1:100). Corresponding secondary antibodies (Alexa Fluor, 1:300, Life Technologies) were diluted in blocking solution for 1 h at RT on a shaker. Counterstaining of nuclei was carried out using 1:2,500 Invitrogen^TM^ Hoechst 33342 (Hoechst; ThermoFisher). All images were acquired using the confocal microscope LSM510 Meta (Zeiss) in combination with the AxioVision V4.6.3.0 software (Zeiss) and further processed with AxioVision software and Photoshop CS6 version 6.2 (Adobe Systems).

### Micro-Electrode Array (MEA) Recordings

Micro-electrode array recordings were conducted using the Maestro system from Axion BioSystems. A 48-well MEA plate with 16 electrodes per well was precoated with 1 mg/ml polyethylenimine (PEI; Sigma Aldrich) for 1 h at RT, washed three times with sterile H_2_0 and air-dried ON in the cell culture hood. Afterward, the plate was pre-coated with Matrigel by placing a 7 μl drop of Matrigel (200 μg/ml) into the center of the well (on top of the electrodes) and incubated for 1 h at 37°C, 5% CO_2_. Remaining coating solution was removed, and 80,000 DNs per well were seeded on the electrodes in each well of the MEA plate by preparing a cell suspension of 7 μl and applying a droplet directly into the center of each well of the MEA plate. Following an incubation of 1 h at 37°C, 5% CO_2_ to ensure settlement of the DNs, 300 μl maturation media (for media formulation see “Differentiation of NPCs and DNs”) was slowly added to each well. Half of the media was refreshed every 2 days. Two weeks after plating, spontaneous activity was recorded at a sampling rate of 12.5 kHz under controlled conditions (37°C and 5% CO_2_) for 10 min on different days. Using Axion Integrated Studio (AxIS 1.4.1.9), a digital low pass filter of 4 kHz and high pass filter of 200 Hz cut-off frequency and a threshold of 5.5x the standard deviation was set to minimize false-positives detections. Raw data was analyzed using the Neural Metric Tool (Axion BioSystems) to analyze spikes. Electrodes that measured more than five spikes per minute were considered active.

### High-Content Imaging (HCI)

High-content imaging-based quantification of MMP and neuronal profiling was assessed using the HCI platform CellInsight CX7 microscope (Thermo Fisher Scientific) and the integrated BioApplications. Briefly, 4- to 8-week-old DNs were split using accutase and seeded at a density of 10,000–20,000 cells/well on Matrigel-coated 96 well plates with black-wall and clear-bottom (μClear, Greiner). The cells were live-stained with non-quenching concentrations of 0.5 nM TMRM (ThermoFisher) for 30 min at 37°C, 5% CO_2_, and counterstained with 1:10,000 Hoechst (Thermo Fisher). Parameters were set as follows: primary object detection (cell nuclei) was based on Hoechst staining, captured in channel 1. Detection of the cellular region of interest, captured in channel 2, was based on TMRM staining which accumulates in active mitochondria as a result of the MMP. Changes in TMRM intensity within the region of interest (ring region with adjusted threshold) were quantified using the “Cellomics CellHealthProfiling v4 BioApplication” (CellInsight CX7, Thermo Fisher Scientific) using the feature “MeanTargetAvgIntensityCh2.” Afterward, neuronal cultures were fixed with 4% PFA for 15 min at RT, stained with TUJ1 antibody and counter-stained with Hoechst (see “Immunostaining” for details on staining method). Parameters were set as follows: primary object detection (cell nuclei) was based on Hoechst staining, captured in channel 1. Detection of the region of interest (neurites) was based on TUJ1 staining, captured in channel 2. The morphological changes of TUJ1-postive cells were quantified using the “Cellomics Neuronal Profiling v4 BioApplication” (CellInsight CX7, Thermo Fisher Scientific). Features obtained with the BioApplication used for evaluation of the neuronal branching complexity were: “NeuriteTotalCountPerNeuronCh2,” “NeuriteTotalLengthPerNeuronCh2,” and “BranchPointTotalCountPerNeuronCh2.” Quantification of apoptosis through Annexin-V intensity was assessed using the HCI platform CellInsight CX7 microscope (Thermo Fisher Scientific) and the integrated BioApplications. Briefly, 4- to 8-week-old DNs were split using accutase and seeded at a density of 10,000–20,000 cells/well on Matrigel-coated 96 well plates with black-wall and clear-bottom (μClear, Greiner). The cells were live-stained with 5 μl Annexin V (protocol from the Annexin V-FITC Kit by MACS Miltenyi was adapted to 2D cell culture) per 10^6^ cells for 30 min at 37°C, 5% CO_2_ and counterstained with 1:10,000 Hoechst (Thermo Fisher). For quantification of the Annexin-V intensity, the TMRM intensity protocol (Cellomics CellHealthProfiling v4 BioApplication) was modified according to the Annexin-V staining.

### Treatment of DNs

FCCP and Ant.A, or Oligomycin were applied to 4- to 8-week-old DNs together with the staining solution (0.5 nM TMRM and 1:10,000 Hoechst) and incubated for 30 min at 37°C, 5% CO_2_. Staining solution containing FCCP and Ant.A, or Oligomycin was removed and DNs were washed three times gently with 1x PBS before corresponding phenolred-free media was applied and cells were measured at the CellInsight CX7. Treatments with ethanol were performed 5 days after re-plating 4- to 8-week-old DNs on black-wall, round bottom 96-well plates (μClear, Greiner). 99.5% ethanol (v/v; Roth) was pre-diluted in culture media as 5 M stock solution freshly before use. Accordingly, 10 mM–2.5 M ethanol was further diluted in phenolred-free culture media and administered to the DNs. For the chronic ethanol exposure, freshly prepared culture media containing ethanol was changed every other day at the same time for 7 days including 1 day of withdrawal. Evaporation of ethanol following daily media changes in unsealed culture plates may provide a pattern of exposure more similar to alcohol consumption observed in human daily heavy drinkers. After 7 days of ethanol exposure, DNs were stained with TMRM and Hoechst (see details above). Following the removal of the staining solution, phenolred-free media containing ethanol was reapplied and incubated again for 1 h before assessment of the MNH at the CellInsight CX7. For the acute ethanol exposure, DNs were first stained with TMRM and Hoechst (see details above) before freshly prepared phenolred-free culture medium containing ethanol was administered to the DNs for 1 h at 37°C, 5% CO_2_. After the incubation time, DNs where immediately processed at the CellInsight CX7. Drug treatment (flavoxate, disulfiram, baclofen and lithium) was performed on 4- to 8-week-old DNs with incubation ON at 37°C, 5% CO_2_. The next day, staining solution containing 0.5 nM TMRM and 1:10,000 Hoechst was added to the media for 30 min at 37°C, 5% CO_2_. Further processing was performed according to the MNH assay as described before.

### Compound Screening – MNH Assay

For the proof-of-concept compound screening, 48 compounds were selected from a library of 700 FDA-approved drugs (Selleckchem- z65122) and 5 drugs were added, used in the context of AUD treatment (Appendix Table 1). Briefly, 10,000–20,000 DNs/well were plated on black-wall, round bottom 96-well plates (μClear, Greiner) 5 days before adding the compounds ON. Compounds were used in a final concentration of 0.5 μM diluted in culture media. DMSO concentration was diluted down below 0.05%. The second day, DNs were stained with 0.5 nM TMRM together with 1:10,000 Hoechst diluted in phenol red-free culture medium for 30 min at 37°C, 5% CO_2_ by adding the staining solution into the media containing the drugs. After removal of the staining solution, compounds together with 1 M ethanol were applied to the DNs and incubated for 1 h at 37°C, 5% CO_2_. Control wells included DNs kept untreated and DNs exposed to 1 M ethanol only. HCI was conducted with the CellInsight CX7 microscope (Thermo Fisher) and analyzed according to the “CellHealth Profiling” and “Neuronal Profiling” BioApplication. The same screening was repeated twice, and the values shown in Figures 6B–D represent the mean values of the two runs (mean, ±SD).

### Statistical Analysis

Data were analyzed using GraphPad-Prism software (Prism 4.0, GraphPad Software, Inc.). Data presentation and respective statistical analysis of each individual graph are described in the respective figure legends. The z-factor is defined as the means (μ) and standard deviations (σ) of both the positive (*p*) and negative (*n*) controls as follows:

$$z-f\,a\,c\,t\,o\,r=1-\frac{3\,\left(\sigma \,p+\sigma \,n\right)}{\left|\mu \,p-\mu \,n\right|}$$

## Data Availability Statement

The raw data supporting the conclusions of this article will be made available by the authors, without undue reservation.

## Ethics Statement

The studies involving human participants were reviewed and approved by the Ethics Committee of the Charité – Universitätsmedizin Berlin (EA1/206/15). The patients/participants provided their written informed consent to participate in this study.

## Author Contributions

JP and AP contributed to the conceptualization. AP, JP, and AZ contributed to the methodology. AZ, JC, NT, and SD contributed to the investigation. AP, JP, and EW contributed to the resources. AZ, AP, and JP contributed to the writing of the original draft. AZ, AP, JP, and AH contributed to the writing – review and editing. AP and JP contributed to the supervision. AZ and AP contributed to the visualization. AP, JP, and AH contributed to the funding acquisition. All authors contributed to the article and approved the submitted version.

## Conflict of Interest

The authors declare that the research was conducted in the absence of any commercial or financial relationships that could be construed as a potential conflict of interest.

## Appendix

**TABLE A1:** List of selected compounds from the FDA-approved drug screening library (z65122).

Compound #	Name	M.w.	Target	Indication	Brief description	CAS number	MMP rel. to UT (value)	Neurite count rel. to UT (value)
1	Bethanechol chloride	196.68	AChR	Neurological disease	Selective muscarinic receptor agonist without any effect on nicotinic receptors	590-63-6	Down (0.750)	Down (0.839)
2	Econazole nitrate (Spectazole)	444.7	Anti-infection, calcium channel	Neurological disease	Ca^2+^ channel blocker, used as an antifungal medicine that fights infections caused by fungus	24169-02-6	Down (0.761)	Down (0.701)
3	Acetanilide (Antifebrin)	135.16	n.d.	Neurological disease	Aniline derivative and has possess analgesic	103-84-4	Down (0.630)	Down (0.761)
4	Cefprozil hydrate (Cefzil)	407.44	n.d.	Neurological disease	Second-generation cephalosporin type antibiotic	121123-17-9	Down (0.737)	Down (0.769)
5	Tolterodine tartrate (Detrol LA)	475.57	AChR	Neurological disease	Tartrate salt of tolterodine that is a competitive muscarinic receptor antagonist	124937-52-6	Down (0.745)	Down (0.780)
6	Azelastine hydrochloride (Astelin)	418.36	Histamine receptor	Neurological disease	Potent, second-generation, selective, histamine antagonist	79307-93-0	Down (0.651)	Down (0.806)
7	5-Aminolevulinic acid hydrochloride	167.59	n.d.	Neurological disease	Intermediate in heme biosynthesis in the body and the universal precursor of tetrapyrroles	5451-09-2	Down (0.697)	Down (0.845)
8	Ronidazole	200.15	Anti-infection	Neurological disease	Antiprotozoal agent	7681-76-7	Down (0.709)	Down (0.799)
9	Miglitol (Glyset)	207.22	Carbohydrate metabolism	Neurological disease	Oral anti-diabetic drug	72432-03-2	Down (0.786)	Normal (0.894)
10	Buflomedil HCl	343.85	Adrenergic receptor	Neurological disease	Vasodilator used to treat claudication or the symptoms of peripheral arterial disease	35543-24-9	Down (0.529)	Up (1.066)
11	PCI-32765 (Ibrutinib)	440.5	Src	Neurological disease	Potent and highly selective Btk inhibitor with	936563-96-1	Down (0.652)	Down (0.814)
12	Amoxicillin (Amoxycillin)	365.4	Anti-infection	Neurological disease	Moderate-spectrum, bacteriolytic, β-lactam antibiotic	26787-78-0	Down (0.619)	Normal (0.909)
13	Cabazitaxel (Jevtana)	835.93	Microtubule associated	Neurological disease	Semi-synthetic derivative of a natural taxoid	183133-96-2	Down (0.768)	Down (0.889)
14	Niclosamide (Niclocide)	327.12	STAT	Neurological disease	Inhibits DNA replication and inhibits STAT	50-65-7	Down (0.728)	Down (0.812)
15	Lurasidone HCl	529.14	Dopamine receptor	Neurological disease	Antipsychotic, inhibits dopamine D2, 5-HT2A, 5-HT7, 5-HT1A and noradrenaline α2C	367514-88-3	Down (0.804)	Down (0.877)
16	D-Phenylalanine	165.19	n.d.	Neurological disease	Carboxypeptidase A, endorphinase and enkephalinase inhibitor, enhances endorphin production and diminishes pain	673-06-3	Down (0.731)	Down (0.871)
17	Palonosetron HCl	332.87	5-HT receptor	Neurological disease	5-HT3 antagonist used in the prevention and treatment of chemotherapy-induced nausea and vomiting	135729-62-3	Down (0.803)	Down (0.811)
18	Azelnidipine	582.65	Calcium channel	Neurological disease	Dihydropyridine calcium channel blocker	123524-52-7	Down (0.682)	Down (0.872)
19	Dexmedetomidine	200.28	Adrenergic receptor	Neurological disease	Sedative medication used by intensive care units and anesthetists	113775-47-6	Down (0.726)	Normal (0.912)
20	Etravirine (TMC125)	435.28	Reverse transcriptase	Neurological disease	Non-nucleoside reverse transcriptase inhibitor (NNRTI) used for the treatment of HIV	269055-15-4	Normal (0.904)	Normal (0.921)
21	Oxybutynin chloride	393.95	AChR	Neurological disease	Anticholinergic medication used to relieve urinary and bladder difficulties	1508-65-2	Down (0.773)	Down (0.833)
22	Tripelennamine HCl	291.82	Histamine receptor	Neurological disease	H1 antagonist, inhibiting PhIP glucuronidation	154-69-8	Up (0.960)	Up (1.146)
23	Articaine HCl	320.84	n.d.	Neurological disease	Dental local anesthetic which contains an additional ester group that is metabolized by esterases in blood and tissue	23964-57-0	Down (0.768)	Normal (0.960)
24	Doxylamine succinate	388.46	Histamine receptor	Neurological disease	Competitively inhibits histamine at H1 receptors with substantial sedative and anticholinergic effects	562-10-7	Down (0.755)	Normal (0.912)
25	Atomoxetine HCl	291.82	5-HT receptor	Neurological disease	Selective norepinephrine (NE) transporter inhibitor with Ki of 5 nM, with 15- and 290-fold lower affinity for human 5-HT and DA transporters	82248-59-7	Normal (0.910)	Normal (0.955)
26	Brinzolamide	383.51	Carbonic anhydrase	Neurological disease	Potent carbonic anhydrase II inhibitor	138890-62-7	Normal (0.850)	Normal (0.934)
27	Ropinirole HCl	296.84	Dopamine receptor	Neurological disease	Selective dopamine D2 receptors inhibitor	91374-20-8	Down (0.791)	Low (0.886)
28	Azlocillin sodium salt	484.48	Anti-infection	Neurological disease	Acylampicillin with a broad spectrum against bacteria	37091-65-9	Up (0.935)	Normal (0.956)
29	Azacyclonol	267.37	5-HT receptor	Neurological disease	Drug used to diminish hallucinations in psychotic individuals	115-46-8	Up (0.955)	Normal (0.940)
30	Reboxetine mesylate	409.5	n.d.	Neurological disease	Norepinephrine reuptake inhibitor	98769-84-7	Normal (0.899)	Down (0.887)
31	Meptazinol HCl	269.81	Opioid receptor	Neurological disease	Opioid analgesic, which inhibits [^3^H]dihydromorphine binding	59263-76-2	Normal (0.891)	Down (0.880)
32	Nalmefene HCl	375.89	Anti-infection	Neurological disease	Naltrexone analog with opioid antagonistic property	58895-64-0	Up (0.964)	Down (0.886)
33	Amidopyrine	231.29	n.d.	Neurological disease	Analgesic, anti-inflammatory, and antipyretic properties	58-15-1	Normal (0.854)	Normal (0.901)
34	Moclobemide	268.74	MAO	Neurological disease	MAO-A (5-HT) inhibitor	71320-77-9	Up (0.952)	Down (0.862)
35	Lithocholic acid	376.57	FXR	Neurological disease	Acts as a detergent to solubilize fats for absorption	434-13-9	Normal (0.859)	Down (0.888)
36	Ethambutol HCl	277.23	Anti-infection	Neurological disease	Bacteriostatic antimycobacterial agent, which obstructs the formation of cell wall by inhibiting arabinosyl transferases	1070-11-7	Down (0.768)	Down (0.875)
37	Acebutolol HCl	372.89	Adrenergic receptor	Neurological disease	β-Adrenergic receptors antagonist used in the treatment of hypertension, angina pectoris and cardiac arrhythmias	34381-68-5	Up (0.943)	Normal (0.957)
38	Hyoscyamine (Daturine)	289.37	AChR	Neurological disease	AChR inhibitor	101-31-5	Normal (0.834)	Down (0.888)
39	Procaine (Novocaine) HCl	272.77	Sodium channel	Neurological disease	Inhibitor of sodium channel, NMDA receptor and nAChR with IC_50_ of 60 μM, 0.296 mM and 45.5 μM, which is also an inhibitor of 5-HT3 with *K*_D_ of 1.7 μM	51-05-8	Normal (0.886)	Down (0.846)
40	Dicyclomine HCl	345.95	M1 and M3 muscarinic receptors antagonist	Neurological disease	Anticholinergic tertiary amine	67-92-5	Normal (0.924)	Down (0.877)
41	Flavoxate HCl	427.92	AChR	Neurological disease	Muscarinic AChR antagonist	3717-88-2	Up (1.018)	Up (1.005)
42	Aclidinium bromide	564.55	AChR	Neurological disease	Human muscarinic AChR M1, M2, M3, M4, and M5	320345-99-1	Down (0.789)	Up (1.050)
43	Bismuth subsalicylate	362.09	COX	Neurological disease	Active ingredient in Pepto-Bismol and inhibits prostaglandin G/H synthase 1/2	14882-18-9	Normal (0.899)	Normal (0.910)
44	Diphemanil methylsulfate	389.51	AChR	Neurological disease	Quaternary ammonium anticholinergic, it binds muscarinic acetylcholine receptors (mAchR)	62-97-5	Up (0.970)	Down (0.881)
45	Doxapram HCl	432.98	TASK-1, TASK-3, TASK-1/TASK-3	Neurological disease	TASK-1, TASK-3, TASK-1/TASK-3 heterodimeric channel function	7081-53-0	Up (0.958)	Down (0.886)
46	Methazolamide	236.27	Carbonic anhydrase	Neurological disease	Carbonic anhydrase inhibitor	554-57-4	Normal (0.885)	Down (0.884)
47	Bextra (valdecoxib)	314.36	COX	Neurological disease	Potent and selective inhibitor of COX-2	181695-72-7	Up (0.952)	Normal (0.898)
48	Primidone (Mysoline)	218.25	Sodium channel	Neurological disease	Anticonvulsant of the pyrimidinedione class	125-33-7	Up (0.961)	Down (0.858)
49	Baclofen	213,661	GABA receptor	AUD drug	Derivative of the neurotransmitter γ-aminobutyric acid (GABA)	1134-47-0	Down (0.631)	Down (0.726)
50	Naltrexone hydrochloride	341.401	Opioid receptor	AUD drug	*N*-cyclopropylmethyl derivative of oxymorphone. Competitive antagonists at the μ-opioid receptor (MOR)	16590-41-3	Down (0.750)	Down (0.790)
51	Acamprosate calcium	181.211	GABA receptor	AUD drug	NMDA receptor antagonist and positive allosteric modulator of GABA_A_ receptors	77337-76-9	Down (0.814)	Down (0.888)
52	Clomethiazole hydrochloride	161.653	GABA receptor	AUD drug	Positive allosteric modulator at the barbiturate/picrotoxin site of the GABA_A_ receptor	533-45-9	Up (0.992)	Normal (0.906)
53	Disulfiram	296.52	Dehydrogenase	AUD drug	Inhibitor of the enzyme acetaldehyde dehydrogenase	97-77-8	Down (0.734)	Normal (0.912)
